# Absorption modes of Möbius strip resonators

**DOI:** 10.1038/s41598-021-88280-x

**Published:** 2021-04-27

**Authors:** Joshua K. Hamilton, Ian R. Hooper, Christopher R. Lawrence

**Affiliations:** 1 Department of Physics and Astronomy, University of Exeter, Exeter, Devon, EX4 4QL UK; 2 QinetiQ Ltd, Cody Technology Park, Farnborough, GU14 0LX UK

**Keywords:** Physics, Applied physics

## Abstract

In this work, the electromagnetic response of a mathematically interesting shape—a Möbius strip—is presented, along with a ring resonator for comparison. Both resonators consist of a central lossy dielectric layer bounded by perfectly conducting layers. For the case of the Möbius strips, the computational results show that there are a family of half-integer wavelength modes within the dielectric layer. These additional modes result in increased absorption, and a corresponding reduction in the radar cross section. Interestingly, rotational scans show that these modes can be excited over a large angular range. This investigation gives an understanding of the electromagnetic response of these structures, paving the way for future experiments on Möbius strip resonators.

## Introduction

The idea behind this work originated from the concept of a Möbius strip (also known as a Möbius loop or band). The discovery of this structure is attributed to the mathematicians Listing and Möbius in 1858^1^. However, it has been shown that Roman mosaics dated ca. 200–250 AD show the concept of these geometries^2^. The Möbius strip is of interest to mathematicians because it is defined to be a surface with only one side and one boundary: for example, if a line is drawn along the edge, it returns to its origin point after two circuits of the loop - a somewhat counter-intuitive result.

A simple visualisation of a Möbius strip can be created by taking a strip of paper, twisting it half a turn, and then taping the ends together. One can move along the total length of the strip without crossing a boundary edge. As a result, this geometry structure has the mathematical property of being non-orientable^1^.

The electromagnetic properties of ring-based resonators have been the focus of a wide range of research efforts. Ring resonators have been used in antenna design^3,4^, frequency selective surfaces^5,6^, metamaterials^7,8^ and more^9^. Resonators created from helically wound dielectric/metal tape have been shown to exhibit interesting electromagnetic properties. These “chiral Swiss roll” structures support a strongly negative refractive index regime above the fundamental resonance^10,11^.

As far as the authors are aware, the majority of published work focused on the electromagnetic properties of Möbius-like structures was conducted by Pond et al.^12–15^. In this work, Möbius resonators were created by twisting a conducting wire and creating a structure with twice the path length when compared with a standard ring of the same radius. As a result, the resonant frequency of the Möbius strip is half that of the standard ring. This could be used to reduce the size of resonators.

The miniaturisation of printed antennas using the concept of a Möbius strip was investigated by Kim et al.^16^. The fabricated antenna resembled a spiral with a “bridge” out-of-plane to link the start and end, creating a pseudo-Möbius geometry. The proposed design showed it was possible to reduce the average radius by 1/3—compared to a classic ring—while keeping the resonance frequency the same. Ballon et al.^17^ investigated the rotational properties of eigenfunctions of Möbius loop topology and their cylindrical ring counterparts. Interestingly, it was shown that the Möbius loop gave rise to half-integral harmonic excitations.

The investigation presented here is focused on ring and Möbius-like resonators that were designed to have a fundamental resonance at 2.5 GHz, as well as additional resonate modes due to the geometry. Since the radar cross section (RCS) is typically used in experiments to investigate the response of these type of resonators, and since this information is currently not available within the literature, this will be the focus of our work. The RCS is a measure of the power scattered in a given direction when an object is illuminated by an incident wave. The RCS is given by the ratio of the power scattered, $P_{\text {s}}$, to the power incident, $P_{\text {i}}$, when measured at the radius, *r*, of the object:

1 $$\begin{aligned} \sigma \equiv \frac{P_{\text {s}}(r)}{P_{\text {i}}(r)} = 4 \pi r^2 \frac{P_{\text {r}}}{P_{\text {i}}} \end{aligned}$$

However, the scattered power is measured at an antenna, $P_{\text {r}}$—the measurement is not located at the object. Therefore, an additional factor is required, as the signal would be greater by $4 \pi r^2$. If the receiver is co-located with the source then the RCS is known as the monostatic RCS. If the receiver is positioned at a different location to the source, this term is known as the bistatic RCS. The scattered power can be influenced by a range of factors that include: the material, the size (relative to the wavelength), the incident angle, and the polarisation of the transmitted and received radiation.

## Results

### Design of the ring and Möbius strip resonators

**Figure 1:**
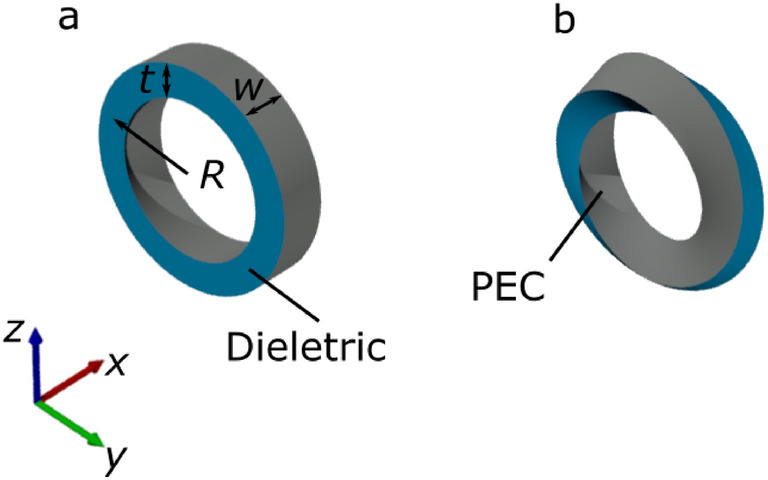
Schematic diagram of (**a**) the ring resonator and (**b**) the Möbius resonator. *R* is the centreline radius, *t* is the thickness of the dielectric, and *w* is the width of the PEC. The blue colouring visualises the dielectric material and the grey visualises the PEC layer.

Two structures of interest were designed within this investigation. The first was a standard dielectric ring with a perfectly electric conducting (PEC) surface on the outer and inner boundaries of the ring (Fig. 1a visualises the PEC as grey and the dielectric material as blue). The second resonator was a dielectric Möbius strip with a PEC layer (Fig. 1b). See Supporting Information for rotational animations of the ring and Möbius geometries.

COMSOL Multiphysics was used for the modelling of these structures. COMSOL Multiphysics is a finite element analysis, solver and multiphysics simulation software. The finite element method (FEM) is a commonly used method for solving computational electromagnetic problems. Our model was based on the benchmark RCS of a metallic sphere model supplied by COMSOL Multiphysics^18,19^. The resonant structure was centred in a spherical modelling domain surrounded by Perfectly Matched Layers (PMLs). The built-in scattered field formulation was used with a time-harmonic background field of the form $\exp (-ik_0x)$. The RCS was subsequently calculated using COMSOL’s built in far-field calculator.

Both the standard metallic ring and the Möbius strip were designed with a radius (*R*)—as measured from the centreline of the strip—of 20 mm, a width (*w*) of 10 mm and a thickness (*t*) of 7 mm. The ring and Möbius strip geometries were designed in a 3D design software (Autodesk AutoCAD) and imported into COMSOL Multiphysics. The inner and outer surfaces of the ring—the same surface for the Möbius strip—were then selected as PEC layers. The thickness of the resonators (i.e. the inner volumes) were given the dielectric constant of $\epsilon = 4.17 - 0.2j$ (mimicking a lossy glass-reinforced epoxy laminate material, known as FR4), leaving the edges between the PEC upper and lower layers exposed.

At GHz frequencies most metals act as exceptionally good conductors with skin depths that are of order $10^5$ times smaller than the wavelength. As such, the vast majority of any absorptive losses will occur in the dielectric spacer material, especially since the electric fields are highly localised within this medium. This allows the use of PEC boundary conditions to mimic the metal coatings, massively reducing the required mesh elements in the model and making the calculations far more tractable.

### RCS of the ring and Möbius strip resonators

It is first useful to understand the simplest system, in this case the ring resonator. The initial investigation focused on the RCS response of two different orientations for two polarisations. The incident plane wave propagated along the x-direction with the two polarisations defined as: E-field in the y-direction ($\text {E}_y = \exp (-ik_0x)$) and z-direction ($\text {E}_z = \exp (-ik_0x)$), respectively. The first orientation—known as ’ring-on’—had the ring orientated in the yz plane, as depicted in Fig. 1. The second-known as ’edge-on’ had the ring orientated in the xz plane.

**Figure 2:**
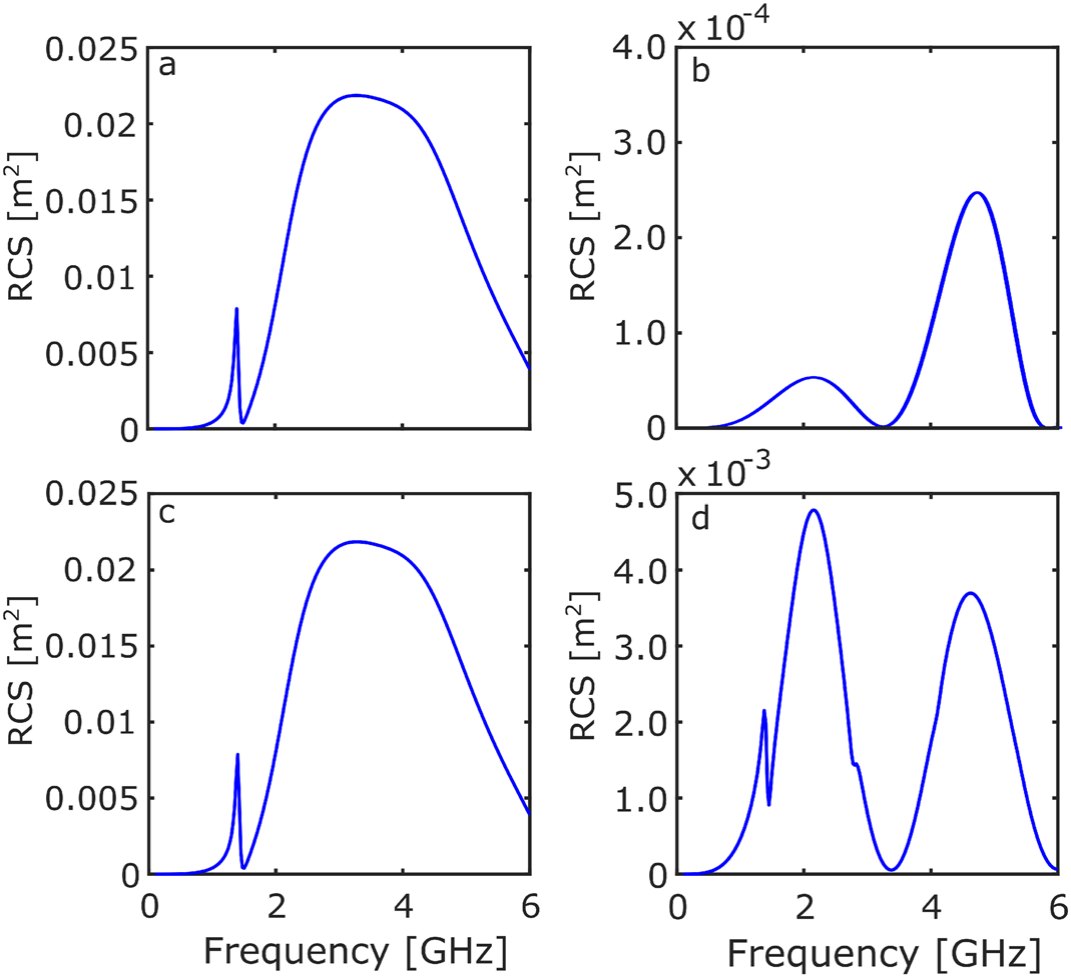
The RCS response of the ring resonator as a function of frequency. (**a**) $\text {E}_y$ illuminating ring-on, (**b**) $\text {E}_y$ illuminating edge-on, (**c**) $\text {E}_z$ illuminating ring-on, and (**d**) $\text {E}_z$ illuminating edge-on.

Figure 2 shows the results of the first investigation. Figure 2a and b show the RCS when the ring resonator is illuminated by a plane wave polarised with $\text {E}_y$ and propagating in the x-direction. The large fundamental mode is visible at 2.5 GHz with an additional mode at 1.4 GHz.

The fundamental resonance of a metallic ring occurs when the wavelength is equal to the circumference, $C_{\text {ring}}=\lambda _{\text {ring}}$. This would be expected to be the same for these PEC coated dielectric structures. However, an additional mode would be expected across the dielectric layer. The dielectric mode corresponds to dipoles present across the thickness of the dielectric. These dipoles are spatially small, so they are poorly coupled to the radiation—the poor coupling resulting in the sharpness of the modes.

The thickness and dielectric loss were chosen to optimise the relative strength and bandwidth of the mode across the dielectric layer, see Supporting Information, Fig. S1. For a ring resonator, integer wavelength quantisations around the dielectric rings are allowed. For the incident radiation to couple to the modes there needs to be a non-zero net dipole moment. In this orientation, summing over the components of the dipoles in the z-direction around the ring sheds light on the allowed coupling of the modes. The only mode with a non-zero net dipole moment is the 1-wavelength mode. All higher order—even and odd—wavelengths have a zero net dipole moment as the top half cancels with the bottom half. As a result of the dielectric properties, there is a sharp increase in RCS at 1.4 GHz, followed by a reduction in the RCS at 1.5 GHz.

Figure 2b shows that the fundamental resonance cannot be excited when illuminated edge-on. The oscillating maxima and minima observed are the result of the specular reflected waves and the back-scattered creeping waves interfering constructively and destructively. This is known as the Mie resonance region and typically occurs when the wavelength is comparable to the structure’s dimension^20^.

Figure 2c shows that, due to rotational symmetry, the RCS response is the same for both polarisations for the ring-on orientation. Figure 2d again shows the Mie resonance region, with a sharp 1.4 GHz mode due to the dielectric layer being excited in this orientation. A second—weakly coupled—dielectric mode can be observed at 2.8 GHz. Due to the combination of orientation and polarisation, the net dipole moment of the 2-wavelength mode is non-zero.

**Figure 3:**
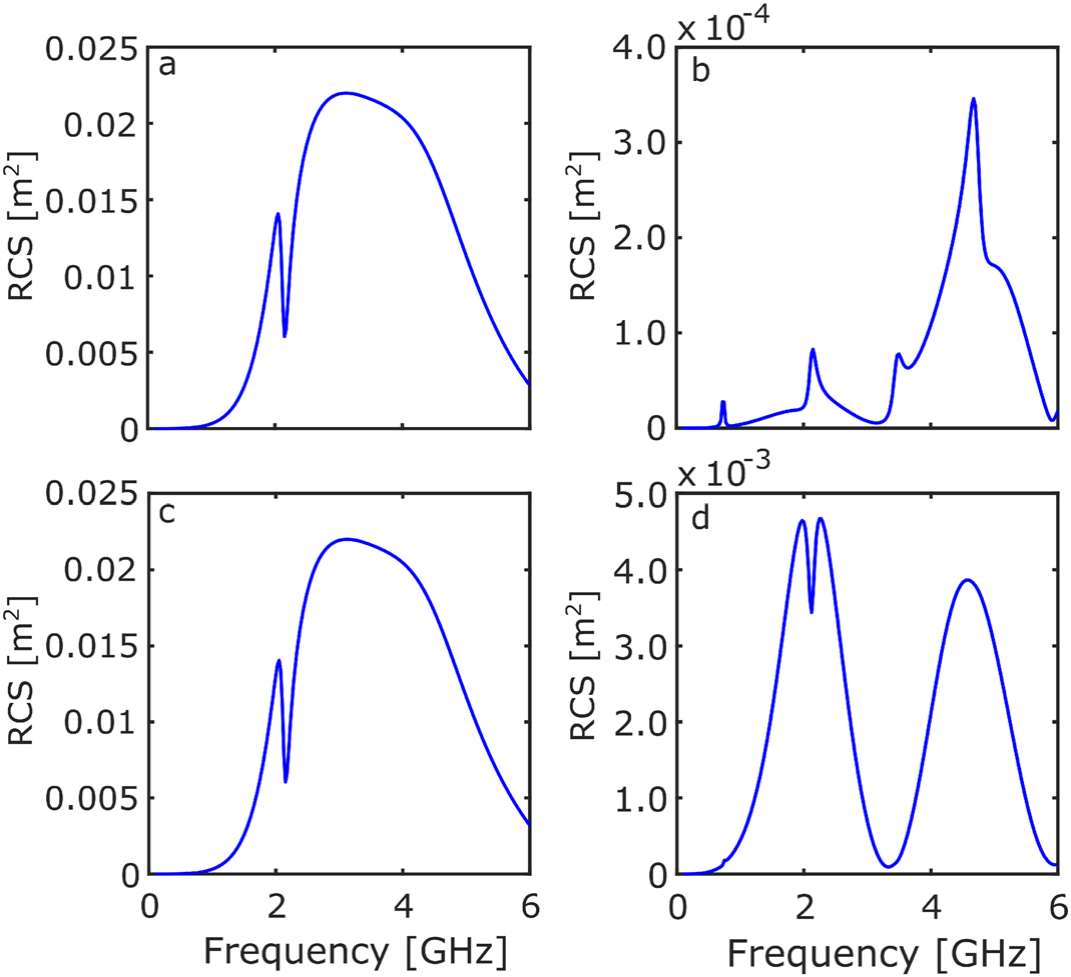
The RCS response of the Möbius resonator as a function of frequency. (**a**) $\text {E}_y$ illuminating ring-on, (**b**) $\text {E}_y$ illuminating edge-on, (**c**) $\text {E}_z$ illuminating ring-on, and (**d**) $\text {E}_z$ illuminating edge-on.

Moving on from the ring resonator, the same analysis was conducted on the Möbius resonator. Figure 3a and b show the RCS when the Möbius resonator is illuminated with a plane wave polarised with $\text {E}_y$ and propagating in the x-direction. Figure 3a shows a similar response to that seen for the ring resonator, the large fundamental mode being unchanged at 2.5 GHz. However, the dielectric mode has shifted in frequency to 2.1 GHz.

Figure 3b shows the edge-on response of the Möbius resonator. Once again, in this orientation the fundamental dipolar mode can not be excited, however four relatively equally spaced resonances are observed at: 0.7 GHz, 2.1 GHz, 3.5 GHz, and 5 GHz.

Figure 3c, d show the RCS when the Möbius resonator is illuminated by $\text {E}_z$ propagating in the x-direction. Figure 3c once again shows that due to rotational symmetry the RCS response is the same for both polarisations for the ring-on orientation. Figure 3d further shows that excitation of the 2.1 GHz mode results in a reduction of the RCS. To understand the origin of the shifting of the dielectric mode, as well as the additional modes, it is useful to inspect the scattering cross section (SCS) and the absorption cross section (ACS).

### SCS and ACS of the ring and Möbius strip resonators

When an electromagnetic wave interacts with an object, that object’s scattering and absorption cross-sections quantify the proportion of the incident radiation that is either scattered or absorbed by the structure^21^.

Similar to the RCS investigation, the ring and Möbius resonators were excited with an incident plane wave propagating along the x-direction with the E-field in the y-direction ($\text {E}_y = \exp (-ik_0x)$) and z-direction ($\text {E}_z = \exp (-ik_0x)$), for the two orientations.

**Figure 4:**
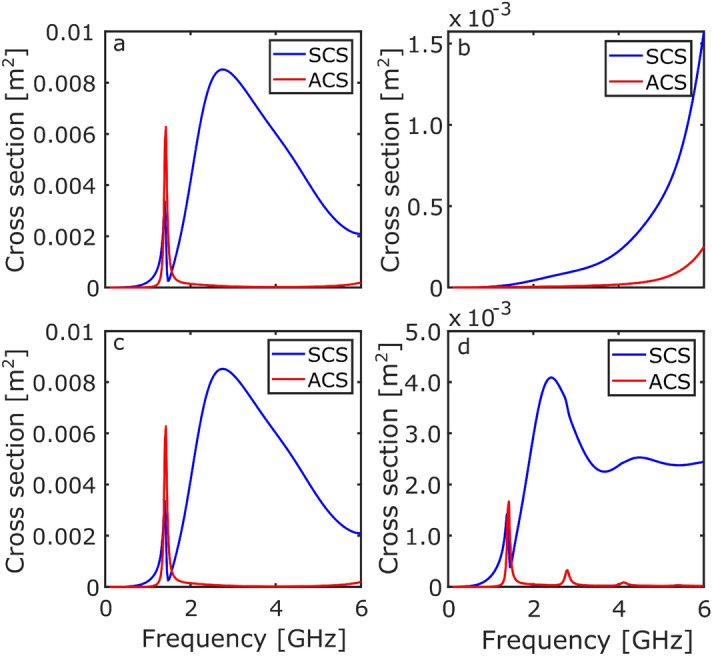
The SCS (blue) and ACS (red) response of the ring resonator as a function of frequency. (**a**) $\text {E}_y$ illuminating ring-on, (**b**) $\text {E}_y$ illuminating edge-on, (**c**) $\text {E}_z$ illuminating ring-on, and (**d**) $\text {E}_z$ illuminating edge-on.

Figure 4 shows the SCS (blue line) and the ACS (red line) for the ring resonator. Figure 4a shows the large fundamental dipolar mode in the SCS response. The spike in both the SCS and ACS is present at 1.4 GHz, due to the coupling to the dielectric mode. This response corresponds to the previously shown RCS (see Fig. 2, and the large absorption is a result of the dielectric loss of the material. Figure 4b shows that when illuminating edge-on the dipolar mode cannot be excited once again. Figure 4c further confirms the rotational symmetry of the ring resonator. An interesting result can be seen in Fig. 4d: additional absorption modes can be observed at 2.6 GHz, 4.0 GHz, and 5.4 GHz. These modes correspond to the higher order wavelength quantisations around the dielectric (2$\lambda$, 3$\lambda$, and 4$\lambda$...).

**Figure 5:**
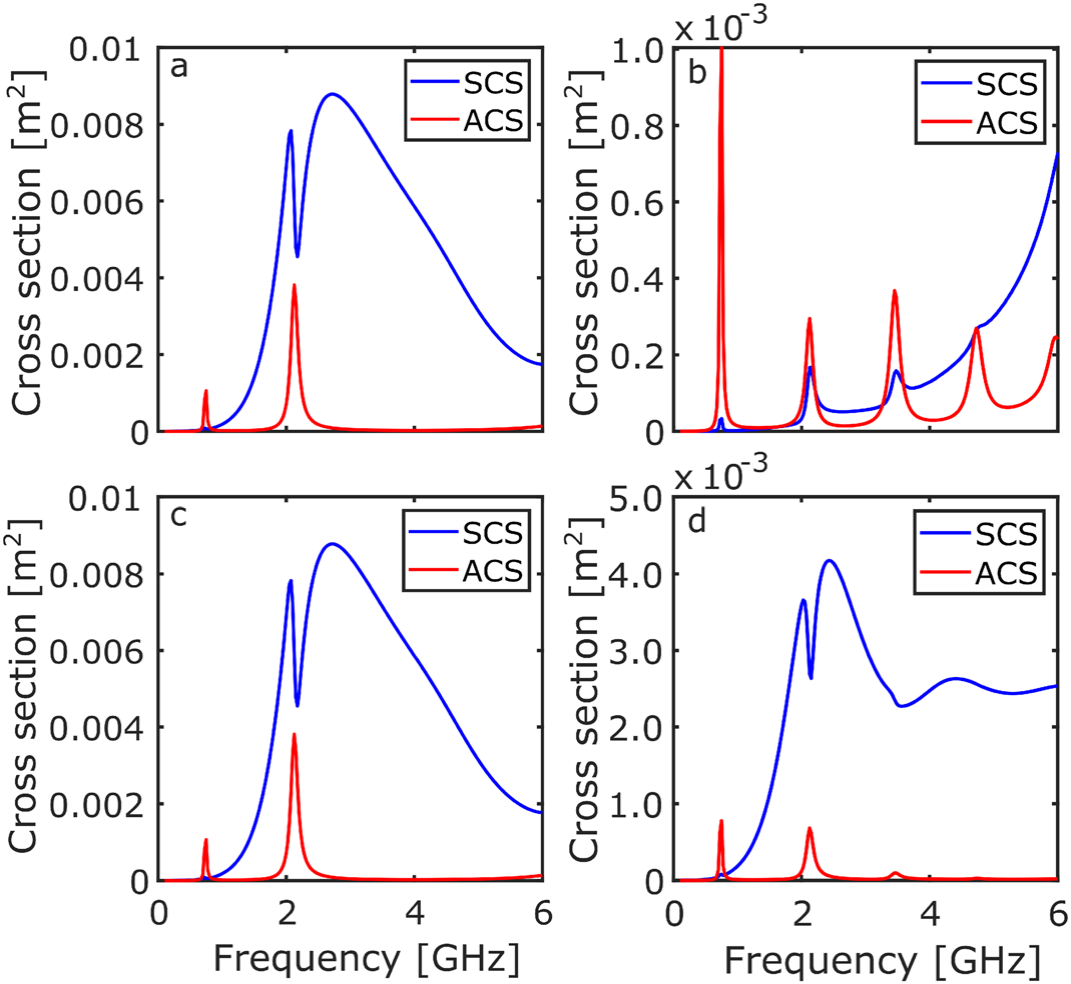
The SCS (blue) and ACS (red) response of the Möbius resonator as a function of frequency. (**a**) $\text {E}_y$ illuminating ring-on, (**b**) $\text {E}_y$ illuminating edge-on, (**c**) $\text {E}_z$ illuminating ring-on, and (**d**) $\text {E}_z$ illuminating edge-on.

Conducting the same analysis with the Möbius resonator reveals additional information regarding the shifting of the dielectric modes. Figure 5 shows the SCS (blue line) and ACS (red line) for the Möbius resonator. Figure 5a shows the 2.1 GHz mode in both the SCS and ACS, as well as the excitation of an absorption mode at 0.7 GHz (previously only visible in Fig. 3b, when illuminating edge-on). Figure 5b clearly shows all the absorption modes present within the structure for the investigated frequency range. The absorption modes are present at: 0.7 GHz, 2.1 GHz, 3.5 GHz, and 5 GHz.

Comparing the absorption modes from the ring resonator (Fig. 4d) and the Möbius resonator (Fig. 5b), the origin of the shifted Möbius modes is clear. When a twist is present, there is a switch between inner to outer charge distributions. As a result, when traversing around the ring and summing over the dipole moments, previously forbidden modes are present and coupling is allowed. These modes correspond to half-integer wavelength quantisations around the dielectric rings—for example, 0.5$\lambda$, 1.5$\lambda$, 2.5$\lambda$, and 3.5$\lambda$.

### Angular response of the ring and Möbius strip resonators

The final investigation in this work was focused on observing how the excitation of the absorption modes changes when the ring or Möbius resonators are rotated relative to the incident radiation. Two rotations were investigated, the first being when the structures are rotated around the z-axis and the second when the structures are rotated around the the x-axis. See Supporting Information for videos of the rotations.

**Figure 6:**
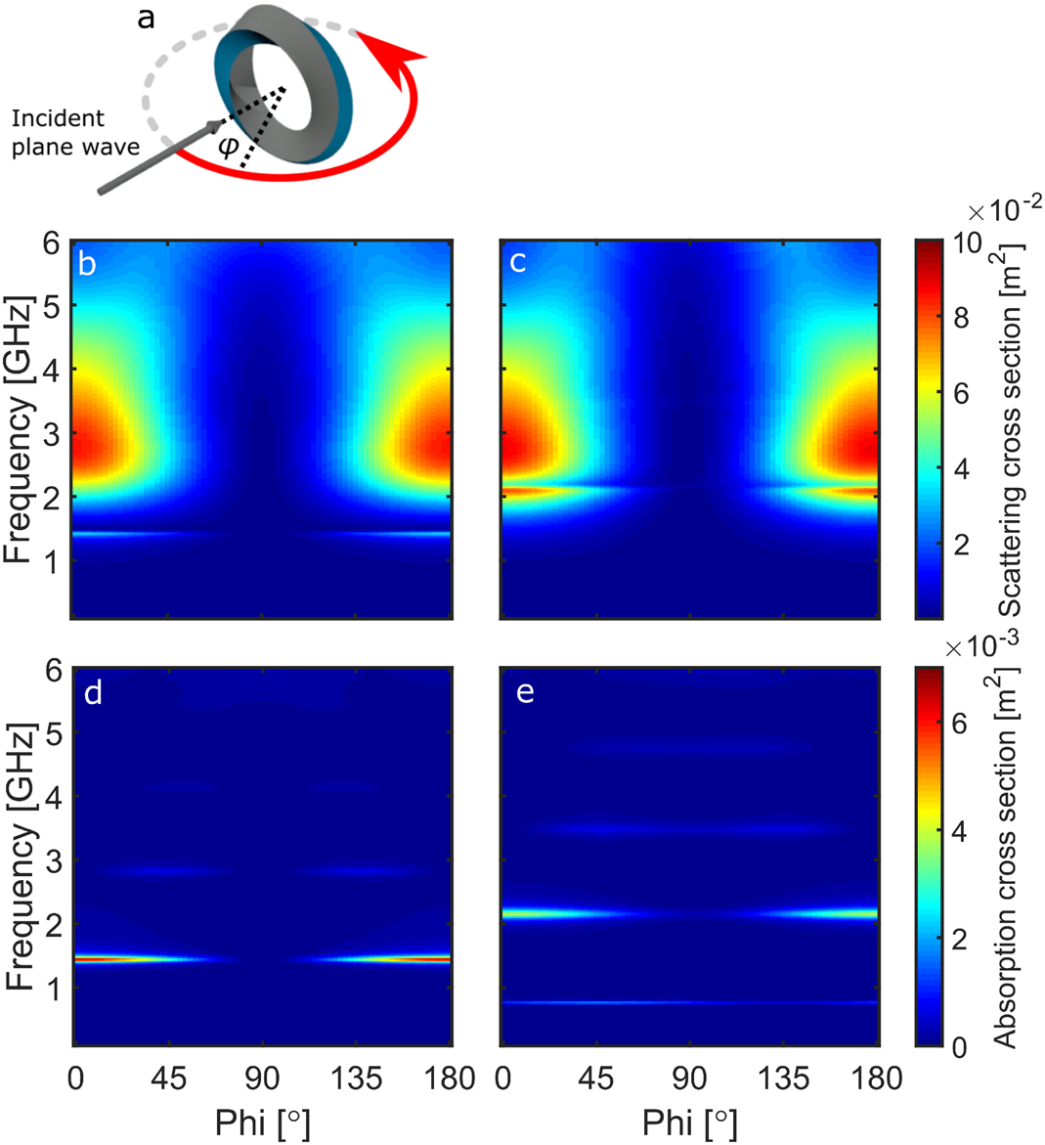
The SCS and ACS response of the ring and Möbius resonators as a function of angle ($\phi$) and frequency. (**a**) Shows a schematic of the rotational plane. The SCS response is shown for $\text {E}_y$ illuminating the (**b**) ring resonator, and (**c**) the Möbius resonator. (**d**) and (**e**) show the corresponding ACS.

Figure 6a shows a schematic of the rotation, the angle $\phi$ being swept through $180^{\circ }$. Figure 6b, c show the SCS for the ring and Möbius resonators, respectively. For both resonators, starting at $0^{\circ }$ (ring-on), the fundamental and dielectric modes are excited, as the angle increases the excitation is decreased until a minimum at $90^{\circ }$ (edge-on). As the angle is increased further, the excitation gradually increases until reaching $180^{\circ }$.

Figure 6d shows the ACS for the ring resonator. There is strong coupling to the 1.4 GHz mode at $0^{\circ }$, which gradually decreases with increasing angle until $90^{\circ }$. The higher order modes at 2.6 GHz and 4.0 GHz are weakly coupled at angles between approximately $10^{\circ }$ and $70^{\circ }$.

Finally, Fig. 6e shows the ACS for the Möbius resonator, for which the lowest order mode (0.5$\lambda$) is excited over the full angle range. There is stronger coupling to the 1.5$\lambda$ mode, with the strength reducing as the angle approaches $90^{\circ }$, which matches the data previously shown in Fig. 5. The higher order Möbius resonator modes are shown to be more angular independent compared to the modes seen in the ring resonator.

The origin of the angular independence can be explained by thinking about the projected area of the geometry relative to the incident radiation. When the Möbius resonator is rotated around the z-axis, there is always dielectric material in the plane of the excitation (See Supporting Information). However, for the ring resonator, at $90^{\circ }$ in Fig. 6d, the orientation is such that there is only PEC in the plane of the excitation, and therefore coupling to the modes is not possible.

**Figure 7:**
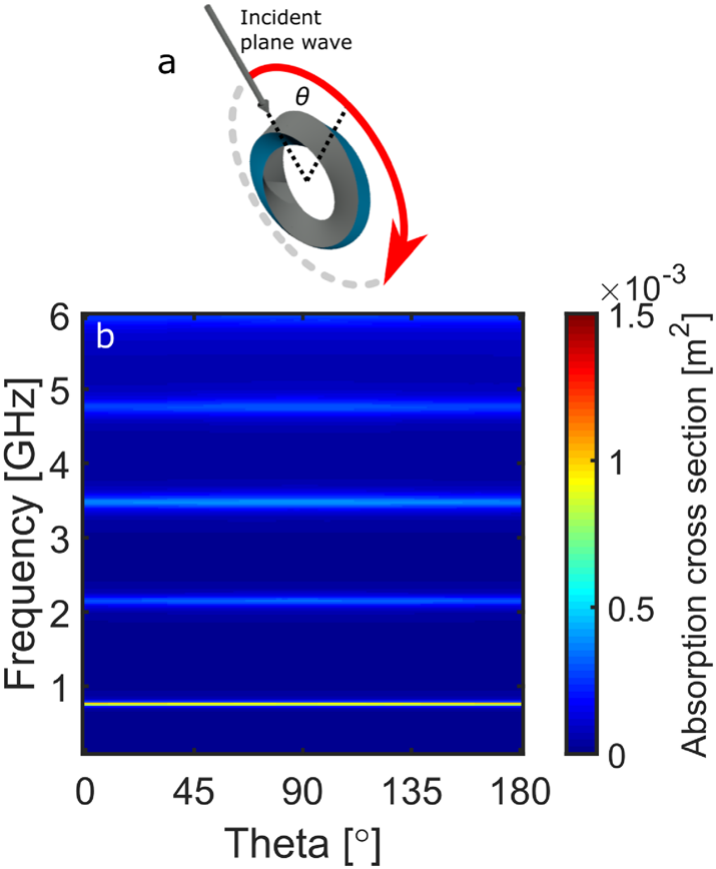
The ACS response of the Möbius resonator as a function of angle ($\theta$) and frequency. (**a**) Shows a schematic of the rotational plane. The response is shown for $\text {E}_y$ illuminating the Möbius resonator edge-on.

Figure 7 shows the ACS when the Möbius resonator is rotated around the x-axis, as well as a schematic of the rotation (Fig. 7a). The dielectric modes supported by the Möbius resonator are completely angle independent when rotated in this orientation. The ring resonator data is not shown for this geometry since the modes are not excited, as can be seen at $90^{\circ }$ on Fig. 6d.

## Conclusion

In this work, the radar cross section of a mathematically interesting shape—a Möbius strip—was investigated, along with a ring resonator for comparison. Both resonators consisted of a central lossy dielectric layer bounded by perfectly conducting layers.

The RCS of both structures was dominated by their fundamental dipolar resonance. However, sharp features were also present due to additional small dipole moments across the thickness of the dielectric core. To more fully understand the response of the resonators, their scattering and absorption cross sections were also calculated. Narrow-band absorption features were evident in the spectra, with those supported by the ring resonator arising from integer wavelength quantisations around the ring, and those for the Mobius resonator arising from half-integer wavelength quantisations. For the case of the Möbius resonator, coupling to these absorption modes is possible over a wide range of polarisations and orientations.

The computational results shown further our understanding of the electromagnetic response of Möbius-like structures. This work will help any future experimental investigations focusing on such structures.

## Supplementary Information

Supplementary Information 1.

Supplementary Information 2.

Supplementary Information 3.

Supplementary Information 4.

Supplementary Information 5.
