# Supplementary material for: Absorption modes of Möbius strip resonators
Source: Sci Rep. 2021 Apr 27;11:9045. doi: 10.1038/s41598-021-88280-x (PMC8079456; doi:10.1038/s41598-021-88280-x)
Supplement: Supplementary file 1 — Supplementary Information 1. [file 41598_2021_88280_MOESM1_ESM.docx]

Supplementary Information

**Absorption Modes of Möbius Strip Resonators**

# Joshua K. Hamilton^1, 2, *^, Ian. R. Hooper^1^, and Christopher R. Lawrence^2^

^1^College of Engineering, Mathematics and Physical Sciences, University of Exeter, Exeter, UK

^1^Department of Physics and Astronomy, University of Exeter, Exeter, Devon, EX4 4QL, UK

^2^QinetiQ Ltd, Cody Technology Park, Farnborough, GU14 0LX, UK

^*^Author for correspondence: J.Hamilton2@exeter.ac.uk

# Movie captions

**Supplementary Movie 1:** The rotation of the ring resonator edge on. The grey regions depict a perfectly electric conducting (PEC) surface and the blue region shows the dielectric core.

**Supplementary Movie 2:** The rotation of the ring resonator ring on. The grey regions depict a perfectly electric conducting (PEC) surface and the blue region shows the dielectric core.

**Supplementary Movie 3:** The rotation of the Möbius resonator edge on. The grey coloured sections depict a perfectly electric conducting (PEC) surface and the blue region shows the dielectric core.

**Supplementary Movie 4:** The rotation of the Möbius resonator ring on. The grey coloured sections depict a perfectly electric conducting (PEC) surface and the blue region shows the dielectric core.

# Supplementary figure

To optimise the coupling to the first resonance due to the dielectric layer (1.4 GHz), thickness and dielectric loss sweeps were run for the ring resonator. The first sweep was the thickness sweep, which swept between 1 mm to 10 mm. Using a thickness of 7 mm, the dielectric loss was swept between 0.6 and 0.2. The aim of these sweeps was to broaden the mode without reducing the depth by a significant amount. For the investigation show in the main text, the dielectric constant was selected as ε = 4.17 – 0.2j.


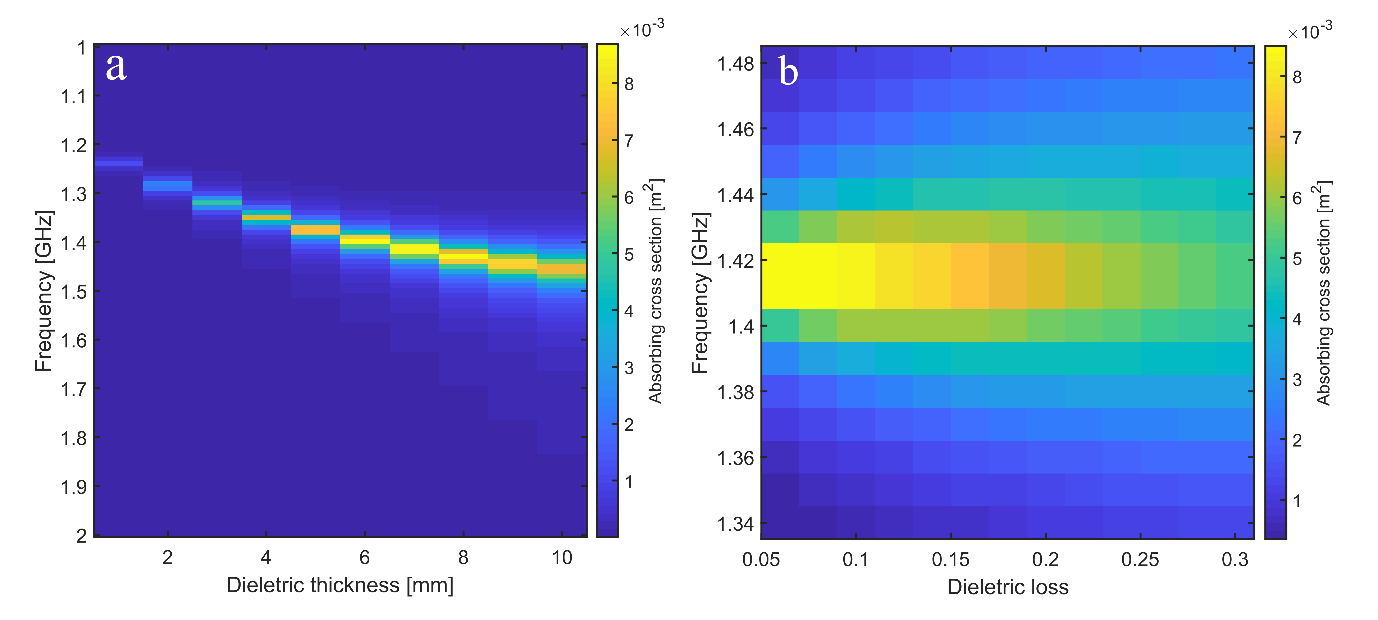


**Supplementary Figure 1.** Absorbing cross sections colour plots for the 1.4 GHz resonance for the ring resonator showing (**a**) the thickness sweep and (**b**) the dielectric loss.
